# A Romance of Charity

**Published:** 1919-11-15

**Authors:** 


### THE FUTURE OF THE BLIND.

### III. A Romance of Charity.

LOUIS BRAILLE's system of tactile reading characters for the use of the blind, which has been amplified but not altered in principle since his era, is based on groups of six or fewer embossed dots to represent each letter or common word, and is capable of being, by means of a simple appliance, also written by the blind themselves. Braille's system was made known to the world by the French philanthropist Valentin Haüy, but it was not until more than half-way through last century that it was adopted in this country and taught to blind persons.

The work of the introduction of Braille literature in England we owe to Dr. Armitage, the founder of the British and Foreign Blind Association, which body commenced its work of publishing books for the blind and of helping them in sundry ways in 1870.

The charity was a semi-private concern which received its chief financial support from the family of Dr. Armitage. The scene of its operations was to be found in a few rooms in the house of the founder, and in a workshop and sundry stores in different parts of London. The work of production of books was at first extremely laborious. In the preparation of the metal plates for printing the first Braille Bible, for instance, every dot was punched separately by hand by a worker who is still in the service of the association, now known as the National Institute for the Blind, and every sheet in each book was printed or pressed by a single manual effort of a workman who operated a hand-lever press.

IMPROVED MACHINERY FOR PRODUCING EMBOSSED BOOKS.

As a contrast to this method of production we are able to compare, in the present workshops in Great Portland Street, the busy stereotyping machines and the power-driven presses which pour forth many hundred sheets per hour.

It was mistakenly stated in a recent number of "St. Dunstan's Magazine" that the National Institute for the Blind (formerly known as the British and Foreign Blind Association) was practically founded by Sir Arthur Pearson and Sir Washington Ranger. This is not so. The Institute, as we shall show later, owes immeasurable thanks to these two gentlemen, but it is to Dr. Armitage that we owe the foundation of this invaluable agency.

The British and Foreign Blind Association continued its work in its confined quarters until early this century, and gathered round it a number of well-known educated blind men and women, amongst whom not the least prominent is the Chairman of the present Executive Council, that eminent blind solicitor Sir Washington Ranger. Mr. Timothy Holmes, the well-known surgeon of St. George's Hospital, who was blind in one eye, was for many years the Chairman of the Association.

In the early years of this century the first paid secretary was engaged in the person of a gentleman who is at present not unknown in the hospital world. He entered upon his duties with much vigour, and his short time of office is marked by the gathering together of the many threads of the Association and the centralisation of the work in Great Portland Street near where Mr. Claude Ferrier's magnificent building, the present headquarters of the National Institute, now stands. After the move to Great Portland Street the work of the Association went forward without a setback, and for some time the late Professor McHardy, of King's College Hospital, was Chairman of the Council and brought many friends to the cause. More recently the undertaking was so fortunate as to attract the interest of Sir Arthur Pearson, the present President, who, in the two years immediately preceeding the war, organised an appeal of a widespread character which attracted large sums to the exchequer. The war intervened, but in a way the intervention helped the cause of the blind generally, through the public attention attracted by the enormous success of St. Dunstan's Hostel (Sir Arthur's own child, as the Institute is his adopted child. The work at St. Dunstan's Hostel for Blinded Sailors and Soldiers was recently described in these columns (THE HOSPITAL, July 19, 1919, p. 407).

BLINDED PEOPLE ARE NOT INDUSTRIAL OUTCASTS.

The war has brought it home to us through the medium of St. Dunstan's that blinded people need not be industrial outcasts. What is needed in every instance is optimism, in them optimism, about them optimism. Sir Arthur Pearson has said "Blindness is only a temporary disability." When he received a new patient into the life of St. Dunstan's he will put his hand on the other's shoulder and say, "Never mind, you and I have seen everything that's worth seeing anyway." He tells the man that he must be taught to be blind, and that there are more than a few occupations that he can follow with as much success as a sighted workman.

The National Institute for the Blind introduces its work into St. Dunstan's during the first days of the new-comer's residence, for each man is at once taught to read and write Braille. Thus a new *entente cordiale* is established between France and England, between the French inventor of embossed printing and the English blinded soldier.

Probably the annual income of the British and Foreign Blind Association during the first thirty years of its existence never exceeded £5,000. The total revenue of the National Institute for the year 1918 was £190,000, or, if we exclude the amounts received for sale of books and other items under trading, nearly £130,000. St. Dunstan's, which is closely allied to the National Institute, had a revenue in the year ending January 31, 1918, of £146,000. It seems almost an incredible romance of charity, but out of the germ of the days of Dr. Armitage should emerge an organisation, or group of two associated organisations, which elicits from the charitable public a third of a million pounds in a single year, and which possesses real estate and investments equal in value to not much less than that sum.

One of the chief points in the policy of the National Institute is co-ordination of charity and several important undertakings such as the Moon Society, the Home Teaching Society, and the Association of Blind Masseuses have come under the wing of the National Institute. The Institute, by means of substantial grants to other organisations of the blind, in addition has obtained considerable influence in the blind world generally of the same kind as that possessed by King Edward's Hospital Fund amongst the hospitals of London. In addition, the Institute is founding or has founded important separate branches such as a home for blind babies and a centre for the higher education of blind girls. Its importance and the scope of its work possess limits which cannot at the present time be seen, and an influence which it is impossible to estimate upon the blind population and those who help them either by charity or through legislation.

**Previous articles appeared on October 18 and 25, p. 72.**

